# Polymer-Enabled Additive Manufacturing for Personalized Drug Delivery and Diagnostic Platforms: Materials, Architectures, Quality Control and Clinical Translation

**DOI:** 10.3390/polym18172053

**Published:** 2026-08-24

**Authors:** Parthiban Pandian, Veeran Sethuraman, Arvind Kumar Shukla, Arulkumar Nagappan

**Affiliations:** 1 Department of Pathology, Saveetha Medical College and Hospital, Saveetha Institute of Medical and Technical Sciences (SIMATS), Chennai 602105, India; dr.parthi2009@gmail.com; 2 Department of Orthopaedics, Saveetha Medical College and Hospital, Saveetha Institute of Medical and Technical Sciences (SIMATS), Chennai 602105, India; 3 School of Biomedical Convergence Engineering, Pusan National University, Yangsan 50612, Republic of Korea

**Keywords:** polymer 3D printing, additive manufacturing, drug delivery, diagnostics, printlets, microneedles, microfluidics, biosensors, hydrogels, photopolymers, quality by design, personalized medicine

## Abstract

Polymer-based three-dimensional (3D) printing has evolved from a prototyping approach toward a manufacturing strategy with emerging clinical relevance for individualized dosage forms, local drug depots, microneedle systems, microfluidic cartridges, biosensor housings and integrated theranostic platforms. Its value arises from the simultaneous control of polymer chemistry, device architecture and process history: infill, porosity, shell thickness, crosslink density, swelling, degradation and surface chemistry can be used as design variables rather than incidental manufacturing outcomes. This review critically synthesizes recent progress in polymer-enabled additive manufacturing for drug delivery and diagnostic applications, with emphasis on thermoplastic and biodegradable polymers, hydrogels, photopolymers, elastomers, conductive composites, stimuli-responsive networks and bioinks. Fused deposition modelling, hot-melt extrusion, semi-solid extrusion, vat photopolymerization, two-photon polymerization, selective laser sintering, binder jetting and inkjet/aerosol jet approaches are compared in relation to drug stability, diagnostic compatibility, feature resolution, scalability and regulatory risk. Particular attention is given to geometry-controlled release, multi-drug printlets, microneedles, implants, scaffold-based local therapy, microfluidic diagnostics, electrochemical biosensors and wearable or closed-loop systems. Translation is discussed through quality-by-design, critical material attributes, critical process parameters, process analytical technology, extractables/leachables, sterilization, point-of-care manufacturing, data integrity and clinical evidence requirements. Future advances should connect polymer–process–property relationships with clinically meaningful use cases, verified quality attributes and realistic regulatory pathways.

## 1. Introduction

Additive manufacturing has expanded approaches for designing dosage forms and diagnostic devices [1,2,3]. Rather than selecting a fixed mould, die or tooling route and subsequently adapting formulation variables to the manufacturing process, 3D printing enables product geometry, material distribution and process history to be designed together. In polymeric systems, this is particularly powerful because the polymer determines not only mechanical shape but also hydration, diffusion, degradation, mucoadhesion, permeability, optical properties, surface adsorption and biological interface. The first approval of a 3D-printed porous levetiracetam product demonstrated that an additively manufactured medicine can satisfy regulatory expectations when the product and control strategy are well defined [4,5]. Since that milestone, research has expanded from oral printlets [6,7], polypills [8,9], pediatric and geriatric dosage forms [10], microneedle patches [11,12], implants and scaffolds [13,14], microfluidic cartridges [15,16], biosensor substrates [17,18], and closed-loop therapeutic-diagnostic platforms [9,19].

The field has now advanced beyond isolated proof-of-concept studies, with increasing attention being given to process capability, printable excipient selection, patient-specific design, analytical validation and decentralized manufacturing [20,21,22]. Nevertheless, the literature remains fragmented across polymer science, pharmaceutical formulation, diagnostic engineering and clinical translation. Therefore, this review focuses specifically on the relationships among polymer properties, printing processes, product architecture and functional performance in drug-delivery and diagnostic applications. Particular attention is given to the material and process trade-offs that influence manufacturability, drug stability, release behaviour, sensing performance, quality control and clinical translation.

### 1.1. Scope and Contribution of This Review

This review focuses on polymer-enabled additive manufacturing for drug-delivery and diagnostic applications, including theranostic platforms that combine sensing and therapeutic functions. Unlike reviews that primarily describe individual polymer classes, printing technologies or dosage forms, the present review examines the interdependence of polymer properties, processing conditions, printed architecture and functional performance. The discussion is guided by four questions: which polymer–printing combinations provide an appropriate balance of printability and biomedical performance; how printing conditions affect the stability of drugs, biomolecules, cells and diagnostic reagents; how printed geometry controls drug release, sensing, sampling and patient interaction; and what quality, regulatory and clinical evidence is required for translation.

The review highlights the recent literature while also retaining seminal studies that are essential for understanding the development of polymer-based 3D printing in drug delivery and diagnostics. Early studies in pharmaceutical additive manufacturing established important design principles, including fused deposition modelling of printlets [3], geometry-controlled drug release [7] and fabrication of multi-drug polypills with programmed release profiles [8]. More recent process-specific studies have examined cold laser sintering [23], direct powder extrusion [24] and selective laser sintering strategies for layer-by-layer dosage design [25]. For diagnostic applications, the discussion draws on the established literature on microfluidic and electrochemical sensors [15,16,17,26], together with recent studies on polymeric microneedles, analytical sensing and diagnostic platforms [12,27,28,29,30]. The recent literature on pharmaceutical 3D printing, clinical translation, personalized medicine and regulatory readiness was also considered to contextualize current developments and identify remaining translational challenges [31,32,33,34,35,36,37,38].

Unlike previous reviews that have primarily organized polymer-based additive manufacturing according to printing technologies, polymer classes, dosage forms or individual biomedical applications, this review presents a material–process–architecture–function framework for understanding translational performance. The central perspective adopted here is that polymer selection is not an isolated material choice but a determinant of printability, structural evolution, drug or biomolecule stability, sensing capability and clinically relevant quality attributes. By connecting molecular and physicochemical characteristics of polymers with processing parameters, printed architectures and final therapeutic or diagnostic outcomes, this review highlights the causal relationships that govern successful translation.

Accordingly, the present review adopts a critical perspective by examining how polymer characteristics, manufacturing parameters and printed architecture collectively influence functional performance, reproducibility and translational potential in drug-delivery and diagnostic applications. This integrated approach considers polymer-enabled additive manufacturing as a translational platform in which material selection, process control, product functionality and clinical requirements must be evaluated together rather than as independent technological components.

### 1.2. Literature Search Strategy

The peer-reviewed literature and regulatory documents were identified through PubMed, publisher databases, journal websites, U.S. Food and Drug Administration (FDA) resources and citation chaining. The literature search was conducted up to June 2026. Search terms were combined using keywords related to “3D printing”, “additive manufacturing”, “polymer”, “drug delivery”, “printlet”, “microneedle”, “hydrogel”, “photopolymerization”, “selective laser sintering”, “direct powder extrusion”, “microfluidics”, “biosensor”, “wearable diagnostic”, “point-of-care” and “quality by design”.

Studies were included when they addressed polymer-based additive manufacturing for drug-delivery systems, diagnostic platforms, biomedical devices, quality-control strategies or translational considerations. Original research articles, relevant reviews and regulatory documents were considered when they provided information related to materials, processes, performance evaluation or clinical translation. Publications unrelated to biomedical applications, studies focused exclusively on non-polymeric systems, and reports lacking sufficient methodological or performance information were excluded.

Priority was given to the literature published from 2021 onward, while earlier studies were retained when they represented foundational concepts, landmark products or technologies that continue to influence current developments. The selected literature was analyzed narratively to identify material–process relationships, functional advantages, limitations and translational challenges rather than to perform a quantitative meta-analysis.

## 2. Polymer Platforms for 3D-Printed Drug Delivery and Diagnostic Systems

Polymer selection represents a critical determinant of the performance and translational success of printed biomedical systems [39]. Although a wide range of polymers can be processed using additive manufacturing technologies, their suitability depends on the balance between processability, drug or biomolecule compatibility, mechanical properties, degradation behaviour and regulatory acceptability [40]. Therefore, polymer platforms are discussed not only based on their printing compatibility but also in relation to how their physicochemical characteristics influence the final therapeutic or diagnostic function [41].

### 2.1. Pharmaceutical Thermoplastics and Biodegradable Polyesters

Thermoplastic polymers are central to fused deposition modelling (FDM), hot-melt extrusion (HME), direct powder extrusion and some powder-bed workflows. Poly(vinyl alcohol), poly(lactic acid), polycaprolactone, ethylene-vinyl acetate, poly(ethylene glycol)-containing blends, cellulose derivatives, pharmaceutical polyvinylpyrrolidone grades and Eudragit-type copolymers have been used as printable matrices or processing aids. FDM and HME have been reviewed as paired technologies for tailored medicines [42], while direct powder extrusion has more recently been positioned as a one-step extrusion pathway that can reduce dependence on preformed filaments [24]. General additive manufacturing materials principles remain relevant because rheology, thermal transitions and mechanical integrity govern whether a feedstock can be printed reproducibly [43].

The processing window of thermoplastic polymers is strongly influenced by molecular weight, glass transition temperature, melting behaviour, crystallinity and melt viscosity. These parameters determine extrusion temperature, polymer flow behaviour, filament formation, interlayer adhesion and potential drug degradation during FDM and HME processing. Therefore, polymer selection for biomedical printing requires consideration of physicochemical properties and processability rather than print compatibility alone. A comparative overview of representative polymers used in FDM- and HME-based biomedical printing is provided in Table 1.

The main translational challenge is balancing printability with drug stability. HME and FDM expose the active pharmaceutical ingredient to heat, shear and residence time. This favours thermally stable small molecules, amorphous solid dispersions and polymers whose melt viscosity can be reduced with compatible plasticizers. Poly(vinyl alcohol) (PVA)-based caplets illustrate how filament formulation and microstructure affect release [6], geometry-controlled tablets show that design alone can alter dissolution at constant composition [7], and broader FDM reviews emphasize that filament diameter, brittleness, thermal degradation and nozzle conditions must be controlled as critical material and process attributes [42]. Direct powder extrusion may simplify feedstock preparation, but it transfers the control problem to powder flow, mixing and residence-time distribution [24]. Therefore, the selection of thermoplastic polymers for pharmaceutical printing requires balancing processing advantages with drug stability, mechanical performance and manufacturing consistency, rather than prioritizing printability alone.

### 2.2. Hydrogels, Natural Polymers and Bioactive Networks

Hydrogels are indispensable for semi-solid extrusion, biofabrication, microneedles, wound-contacting systems and biomolecule-compatible delivery. Alginate, gelatin, chitosan, hyaluronic acid, cellulose derivatives, polyethylene glycol diacrylate, polyacrylamide derivatives and peptide-based networks provide hydrated matrices in which diffusion, swelling, degradation and bioadhesion can be tuned. Hydrogel design principles for controlled delivery are well established [56], while extrusion-bioprinting papers emphasize the need for shear-thinning recovery, crosslinking control and cytocompatibility [57,58,59].

The crosslinking mechanism represents a critical determinant of hydrogel performance because it governs network architecture, mechanical properties, swelling behaviour and molecular transport. Ionic crosslinking, such as calcium-mediated gelation of alginate, enables rapid gel formation under mild conditions, although the resulting network properties may differ from covalently crosslinked systems in terms of mechanical strength, stability and responsiveness [56,58]. Covalent crosslinking approaches, including photoinduced polymerization of methacrylated polymers such as PEGDA, provide greater control over crosslink density, elastic modulus and structural stability; however, excessive network formation may restrict swelling, molecular diffusion and cellular interaction [57,59]. Thermally responsive and stimuli-sensitive hydrogels provide temperature-dependent or externally regulated gelation behaviour, but their mechanical properties, degradation behaviour and shape retention require careful optimization for extrusion-based printing [60,61,62,63]. Therefore, hydrogel selection for biomedical additive manufacturing requires balancing crosslinking kinetics, network stiffness, degradation characteristics, diffusion properties and print fidelity rather than considering printability alone [56,59,63].

For tissue-facing regenerative applications, engineered biomaterials must also support in situ repair and biological integration rather than merely carry a drug payload [60].

Recent advances have further expanded hydrogel-based printing through the development of bioactive, stimuli-responsive and hybrid polymer networks that improve print fidelity, biological compatibility and controlled therapeutic delivery. These systems highlight the transition of hydrogels from passive drug carriers toward multifunctional platforms capable of integrating structural support, biological signalling and responsive release behaviour [61,62,63,64].

Stimuli-responsive hydrogels add a temporal dimension to polymer design. pH-sensitive networks can exploit gastrointestinal, wound or tumour microenvironment gradients; thermoresponsive systems can undergo in situ gelation; enzyme-responsive systems can link release to pathology; and electroresponsive or magnetic composites can permit externally triggered release [56,59,60]. Recent magnetic nanocomposite hydrogel systems have demonstrated that nanoparticle incorporation can improve rheological behaviour, enhance thixotropic properties and enable magnetic field-driven diffusion-controlled drug release, expanding the functional capabilities of responsive polymer networks [65]. In addition, nanomaterial-based platforms are increasingly being investigated for integrated drug delivery, bio-imaging and diagnostic applications, although their translation requires careful consideration of safety, reproducibility and biological compatibility [66]. A clinically useful responsive system should not be judged solely by its material sophistication or responsiveness. Its value should be determined by whether the polymer network provides a measurable therapeutic or analytical advantage over conventional passive systems while maintaining manufacturability, sterilizability, chemical stability and reproducible performance.

### 2.3. Photopolymers, Elastomers and Vat-Printable Resins

Stereolithography, digital light processing, continuous liquid interface production, two-photon polymerization and volumetric printing rely on photochemical conversion of liquid resins into solid networks. These methods provide high resolution and smooth surfaces, making them well suited for microneedles, microfluidics, optical components, micro-implants and intricate drug-eluting lattices [67,68,69,70,71]. Common chemistries include acrylates, methacrylates, poly (ethylene glycol) (PEG)-based networks, urethane acrylates and thiol-ene systems. The advantage of high spatial resolution is balanced by the need to quantify photoconversion, residual monomer, photoinitiator, degradation products and extractables, particularly because many commercial resins were designed for prototyping rather than drug contact or diagnostic assays [69,70]. Therefore, the suitability of photopolymer systems for biomedical applications should be determined not only by printing resolution but also by chemical safety, long-term stability, biological compatibility and regulatory acceptability.

Elastomers such as silicones, thermoplastic polyurethanes and soft acrylate networks are important for wearable diagnostics, flexible reservoirs, conformal patches, valves and microfluidic seals. Their compliance can improve skin contact and reduce mechanical mismatch with tissue [72]. However, elastomers can adsorb hydrophobic drugs and assay reagents, exhibit extractable profiles that vary with curing, and require robust interfacial bonding in multi-material devices.

### 2.4. Conductive, Magnetic and Nanocomposite Polymers

Diagnostic devices frequently require conductive pathways, electrochemical electrodes, optical contrast, magnetic manipulation or catalytic activity. Polymer composites containing carbon black, graphene, carbon nanotubes, metallic nanoparticles, MXenes, magnetic nanoparticles or intrinsically conductive polymers can be formulated as printable filaments, pastes or inks. Early electrochemical 3D-printing reviews established the value of printed electrodes and cells [17], while later analytical reviews stressed that the material itself is a determinant of sensitivity, fouling and reproducibility [73]. Additive-manufacturable biosensor-electrode materials are particularly relevant where a polymer must provide both processability and electrochemical function [74].

Patient-facing accessibility examples, such as Braille and Moon-patterned printlets, also show how polymer printing can add user-centred function beyond drug release [7]. However, multifunctional polymer composites introduce additional challenges because improvements in conductivity, magnetic responsiveness or sensing capability may be accompanied by issues related to filler dispersion, reproducibility, cytotoxicity and regulatory acceptance. Therefore, the future development of conductive and nanocomposite polymers should focus on achieving a balance between enhanced functionality and reliable biomedical performance. Table 2 compares the principal polymer classes used in polymer-based 3D printing and outlines their relevance to drug delivery and diagnostics, with emphasis on material performance, biomedical applicability, regulatory acceptability, and translational challenges.

## 3. Printing Technologies and Process-Product Relationships

**Figure 1:**
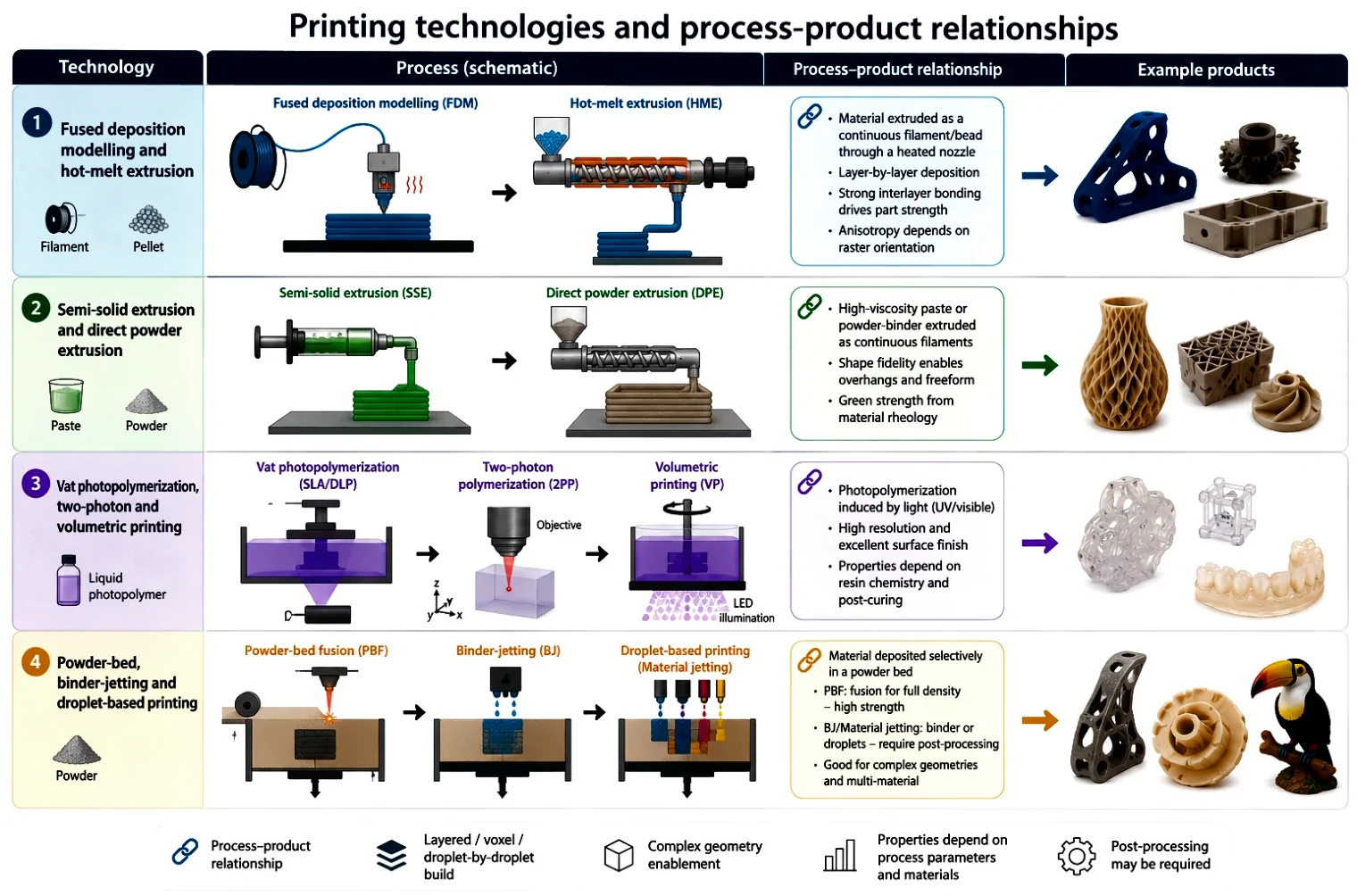
Process–product relationships in polymer-based 3D printing technologies for biomedical applications. Created with BioRender.com.

The major printing technologies and their process–product relationships are illustrated in Figure 1, created with BioRender.com (accessed on 25 May 2026). These include fused deposition modelling and hot-melt extrusion, semi-solid extrusion and direct powder extrusion, vat photopolymerization, two-photon and volumetric printing, as well as powder-bed, binder-jetting, and droplet-based printing. The different material deposition and solidification mechanisms used by each technique have a substantial impact on the final products’ physicochemical characteristics, drug loading capacity, printing resolution and structural integrity, all of which determine their suitability for pharmaceutical and biomedical applications [52,86]. However, no single printing technology provides an optimal solution for all applications; instead, technology selection requires balancing material compatibility, drug or biomolecule stability, production scalability, quality control requirements and the intended clinical use.

### 3.1. Fused Deposition Modelling and Hot-Melt Extrusion

FDM is widely studied because it is accessible, digitally programmable and compatible with thermoplastic pharmaceutical polymers. In a typical pharmaceutical workflow, a drug-loaded filament is first prepared by HME and then printed by FDM. The formulation must satisfy both pharmaceutical and mechanical requirements: drug content and stability must be acceptable, while filament diameter, stiffness and melt viscosity must support reproducible feeding and deposition [6,7,8,42]. Architecture is a powerful design variable. Changing infill density, strand spacing, shell thickness and tablet geometry can alter disintegration and dissolution without changing composition printlets [6,7]. For diagnostic applications, FDM is useful for housings, holders, cartridges and rapid prototyping. Its limitations are surface roughness, layer-line porosity and limited feature size relative to capillary microfluidics.

Hybrid manufacturing can overcome some limitations of FDM by combining its advantages in rapid, low-cost fabrication of mechanically robust structures with complementary technologies that provide higher resolution, specialized surface properties or functional components [87,88]. For biomedical applications, hybrid strategies may integrate FDM-printed polymer frameworks with microfluidic membranes, conductive electrodes, photopolymerized microstructures, drug-loaded hydrogels or soft lithographic elements. Such combinations enable functions that are difficult to achieve using a single printing method, including integrated drug release and sensing, improved fluid handling, enhanced structural precision and multifunctional therapeutic platforms.

Interfacial adhesion is a critical factor in hybrid polymer printing because dissimilar materials often exhibit differences in surface energy, chemical functionality and mechanical behaviour. For example, hydrophobic thermoplastics such as PLA generally show limited affinity toward hydrophilic hydrogel networks, requiring strategies that improve interfacial compatibility. Adhesion between thermoplastic frameworks and hydrogel phases can be enhanced through mechanical interlocking generated by surface roughness and porous architectures, hydrogen bonding between functional groups, surface activation methods and the formation of interpenetrating polymer networks. Similarly, integration of conductive electrodes with polymer substrates requires control of polymer–filler interactions, surface modification and electrical continuity across the interface. Therefore, hybrid additive manufacturing requires optimization of interfacial chemistry and mechanical coupling in addition to printing parameters to achieve stable multifunctional biomedical constructs [23,43,83]. However, hybrid manufacturing also introduces additional challenges related to material compatibility, interface bonding, process synchronization, sterilization and quality control [89]. However, its translational value depends on whether the combination of manufacturing approaches provides measurable improvements in product performance, reproducibility and scalability compared with optimizing a single printing technology [78,90]. Therefore, hybrid strategies should be considered as application-driven solutions rather than universal replacements for conventional additive manufacturing workflows.

### 3.2. Semi-Solid Extrusion and Direct Powder Extrusion

Semi-solid extrusion prints pastes, gels and viscous inks at low temperature. It is attractive for thermolabile drugs, biologics, high drug loading, multi-material polypills and cell-laden constructs. The five-in-one polypill demonstrated independent immediate and sustained-release compartments within a single printed dosage form [8], while related multi-drug tablets showed that compartment position and formulation can separate release profiles [9]. For hydrogel and bioink systems, printability depends on shear-thinning behaviour, recovery after deposition and crosslinking kinetics rather than only on drug loading [59].

Direct powder extrusion (DPE) is a more recent extrusion-based approach that can reduce the need for pre-prepared filaments. Systematic reviews highlight its potential for personalized drug delivery, but also the need to understand powder feeding, mixing, residence time, screw design and die swell [25]. In direct powder extrusion, polymer molecular characteristics become particularly important because of the absence of a preformed filament transfers control of material behaviour directly to the extrusion process. Molecular weight and polydispersity influence chain entanglement, melt viscosity and flow stability, thereby affecting feeding consistency, die swell and strand formation [24,44]. Polymers with higher molecular weight may provide improved mechanical integrity but can increase melt viscosity and extrusion resistance, whereas broader molecular weight distributions may introduce variability in flow behaviour and printed structure [44]. In addition, thermal transitions, crystallinity and polymer–drug interactions determine the processing window, thermal stability and final product performance [24]. Powder flow behaviour, segregation and feeding variability remain additional challenges in filament-free extrusion systems, particularly for maintaining dose accuracy and content uniformity [91,92]. Therefore, successful DPE requires optimization of polymer molecular architecture, powder characteristics and extrusion parameters rather than relying only on geometric control of the printed dosage form.

### 3.3. Vat Photopolymerization, Two-Photon and Volumetric Printing

Vat photopolymerization offers the resolution needed for microneedles, organ-on-chip devices, optical diagnostics and complex drug-eluting microstructures. Recent reviews on photopolymerization-based drug delivery emphasize oral formulations, microneedles, implantable devices, microrobots and emerging smart systems [68]. Microfluidic studies demonstrate that resin selection, post-curing, ageing and material characterization are essential because small amounts of leachable photochemistry can interfere with capillary flow, enzymes, cells or immunoassays [16,27,28,30,69,70]. Two-photon polymerization enables microscale or nanoscale polymer features, including high-aspect-ratio microneedles and microdevices. Volumetric printing promises rapid fabrication without layer-by-layer stepping, potentially reducing stair-stepping and enabling soft hydrogel constructs [79,80].

These approaches are promising but still require stronger evidence on throughput, resin safety, sterilization and quality control before routine pharmaceutical or diagnostic use. Although vat photopolymerization provides exceptional control over microarchitecture and surface features, the clinical relevance of this advantage depends on overcoming challenges associated with resin biocompatibility, residual chemical components, manufacturing scalability and long-term product stability [81,82]. Therefore, future development should focus on balancing fabrication precision with materials safety and regulatory requirements.

### 3.4. Powder-Bed, Binder-Jetting and Droplet-Based Printing

Selective laser sintering avoids filament preparation and can generate porous pharmaceutical structures. It has been explored for orally disintegrating printlets, multi-layer tablets and powder-based personalized medicine. Cold laser sintering was proposed as a lower-energy route for pharmaceutical selective laser sintering (SLS) [23], while layered drug–polymer SLS was recently investigated as a simplified strategy for individually dosed tablets [25]. Binder jetting can produce highly porous rapidly dispersing dosage forms, with Spritam representing a commercial example demonstrating that a powder-based 3D-printed oral product can achieve regulatory approval when product and process controls are adequately established [4,5]. Inkjet, aerosol jet and related droplet technologies deposit small volumes of drug, reagent or conductive ink. They are especially relevant for biosensor fabrication, reagent patterning and thin-film combinatorial dosing. The formulation window is narrow: viscosity, surface tension, particle size, evaporation rate and bioactivity must all be maintained. Nozzle clogging, coffee-ring deposition and drying gradients can undermine uniformity.

As shown in Table 3, each 3D printing technology offers distinct biomedical advantages but also presents specific quality risks related to material compatibility, dose uniformity, resolution, mechanical integrity, sterility and reproducibility. Therefore, technology selection should be guided by the intended clinical application rather than the technical capability of the printer alone. Powder-based and droplet-based approaches provide opportunities for rapid, flexible manufacturing; however, their broader adoption will depend on overcoming challenges related to feedstock consistency, process control, scale-up and validation of final product performance [93,94].

Although printing technology determines material processing conditions and product architecture, the clinical relevance of polymer-based additive manufacturing is ultimately defined by its ability to improve therapeutic delivery, diagnostic performance and patient-specific healthcare solutions [97,98]. Therefore, subsequent sections focus on application-oriented evidence, including drug-delivery systems, diagnostic platforms, quality control strategies and translational challenges.

## 4. Drug Delivery Applications Enabled by Polymer-Based 3D Printing

While polymer selection and printing processes determine the technical feasibility of additive manufacturing, the ultimate value of these systems is determined by their ability to address clinically relevant challenges [99,100]. Therefore, drug-delivery applications are discussed from a translational perspective, considering not only fabrication capability but also therapeutic advantage, dose control, patient-specific requirements and evidence needed for clinical adoption.

### 4.1. Geometry-Controlled Release and Digital Dose Personalization

In printed medicines, geometry becomes a pharmaceutical variable. Porosity, infill, strand diameter, shell count, channel network and surface-area-to-volume ratio determine hydration, diffusion length and disintegration. These architectural parameters must be evaluated together with physicochemical characteristics such as mechanical strength, polymer degradation behaviour, drug distribution and dissolution kinetics because changes in internal structure can influence both release performance and product robustness [45,48]. Seminal work showed that tablet geometry and internal architecture can modulate release even when the formulation is unchanged printlets [6,7]. These studies represent an important shift from conventional dosage-form development, where release characteristics are primarily controlled through formulation composition, toward digitally defined architectures where geometry becomes an additional pharmaceutical variable [101,102]. However, unlike conventional tablets manufactured under highly standardized processes, printed dosage forms require validation of the relationship between digital design parameters, physical characteristics and dissolution performance to ensure reproducible therapeutic outcomes. More recent studies extend the concept to SLS, DPE, photopolymerization and multi-material systems [23,24,25,68]. Personalized dose is not the same as personalized therapy. Dose variability must be clinically meaningful, manufacturable and verifiable. A robust system should link a digital prescription or design file to a validated design space: file parameters should predict dose, mass, dimensions, mechanical properties and dissolution. For narrow-therapeutic-index drugs, regulatory tolerance for variation will be low, making in-line or at-line content verification essential.

Although geometry-controlled drug release represents one of the major advantages of additive manufacturing, its clinical value depends on the ability to translate digital design parameters into predictable therapeutic outcomes. Compared with conventional dosage forms, printed systems provide greater flexibility in modifying dose, internal architecture and release profiles; however, this advantage introduces additional quality requirements because small variations in printing parameters may alter porosity, mass distribution and dissolution behaviour [98,103,104]. Therefore, future development should focus on establishing validated relationships between design parameters, critical quality attributes and in vivo performance rather than relying solely on geometric customization as a measure of personalization. Compared with conventional tablets, which generally rely on formulation composition and fixed manufacturing processes to achieve controlled release, printed dosage forms provide an additional design variable through spatial control of internal architecture. However, this advantage is clinically meaningful only when geometric customization results in improved dose individualization, therapeutic control or patient outcomes that cannot be achieved efficiently through conventional approaches.

### 4.2. Multi-Drug Printlets and Polypills

Polypharmacy is a major driver for printed polypills. Multi-compartment printing can place immediate-release drugs in rapidly accessible regions and sustained-release drugs in diffusion-limited compartments. The early five-in-one polypill and related multi-drug studies demonstrated how extrusion printing can combine multiple release profiles in one object [8,9]. This example illustrates the potential advantage of additive manufacturing over conventional fixed-dose combinations by enabling spatial separation of active ingredients and independent control of release behaviour within a single dosage form. However, the clinical benefit of such systems must be demonstrated through improved therapeutic management rather than simply increased formulation complexity. The translational value lies in reducing pill burden, improving adherence, supporting individualized regimens and reducing dosing errors. However, multi-drug printing also increases formulation complexity. Drug–drug compatibility, drug–excipient interactions, stability, dose flexibility and clinical governance must be resolved. A printed polypill should be pursued when it addresses a real therapeutic challenge, not simply because multiple drugs can be co-printed.

In multicomponent printed systems, the polymer matrix acts as a physical and chemical barrier that can influence drug migration between adjacent compartments. Diffusion of active substances is governed by polymer free volume, chain mobility, glass transition behaviour, matrix hydration and drug–polymer interactions; therefore, polymers with lower molecular mobility or stronger drug–polymer affinity may reduce unwanted intercompartmental diffusion [103,104]. During FDM-based processing, thermal exposure may increase molecular mobility and potentially promote drug redistribution, whereas in semi-solid extrusion systems, water content, swelling behaviour and crosslink density can influence diffusion pathways within hydrogel-based compartments [52,63]. Storage stability further depends on polymer relaxation, moisture uptake, phase separation and changes in the physical state of the incorporated drugs, requiring evaluation of compartment integrity over time [105,106]. Therefore, successful development of printed polypills requires not only spatial separation of active ingredients but also rational selection of polymer matrices that provide adequate diffusion control and long-term physicochemical stability.

### 4.3. Microneedles and Transdermal Platforms

Polymeric microneedles represent one of the more advanced application areas for high-resolution polymer printing. They can bypass gastrointestinal degradation, improve patient acceptability, deliver vaccines or biologics and access interstitial fluid for minimally invasive diagnostics. Direct 3D printing and printed-master micromoulding have produced insulin-delivery microneedles [107], anticancer microneedles [108], undercut vaccination systems [109], diabetes therapy patches [110], biomarker extraction devices [68] and remote-controlled hollow microneedle platforms [9]. Razzaghi provides a detailed perspective on non-transdermal microneedle applications [12], whereas this review positions microneedles as one component within the broader drug-delivery and diagnostic manufacturing landscape. Foundational microneedle safety and vaccine-delivery principles are retained to support the translational discussion [111,112]. The critical tradeoff is mechanical strength versus dissolution or swelling. Needles must penetrate skin without fracture, but the polymer must dissolve, swell or permit transport at a clinically useful rate. Photopolymerization, two-photon polymerization and printed moulds have all been used. For direct photopolymerized microneedles, residual monomers and photoinitiators demand rigorous extraction and biocompatibility testing.

Recent advances in polymeric microneedle fabrication have focused on improving mechanical strength, dimensional precision and drug loading capacity through advanced polymer systems and high-resolution additive manufacturing approaches [12,75]. These developments indicate a transition from proof-of-concept microneedle devices toward clinically relevant platforms; however, clinical translation requires systematic evaluation of insertion performance, needle geometry, tip sharpness and fracture resistance to ensure reliable skin penetration and mechanical integrity [12,29]. In addition, drug-content uniformity, dissolution or swelling behaviour, storage stability and manufacturing reproducibility must be validated to ensure consistent therapeutic or diagnostic performance under practical use conditions [71,75].

### 4.4. Implants, Scaffolds and Local Therapy

Scaffold architecture is a critical determinant of degradation behaviour because pore size, porosity and interconnectivity regulate fluid penetration, surface area exposure and polymer hydrolysis. In printed polymer scaffolds, pore sizes are commonly designed within the range of approximately 100–500 µm, while total porosity is often controlled between 50 and 90% depending on the intended tissue-engineering application; these architectural parameters influence nutrient transport, mechanical stability and degradation kinetics. Larger and highly interconnected pores generally enhance water diffusion and increase the accessible surface area, which can accelerate degradation and mass loss, whereas lower porosity structures may restrict fluid transport and delay polymer erosion [113,114]. The degradation mechanism also depends on polymer chemistry. PCL generally undergoes slow hydrolytic degradation because of its hydrophobic nature and semicrystalline structure, whereas PLA degradation is influenced by ester hydrolysis, crystallinity and autocatalytic accumulation of acidic degradation products within the matrix [39,76,77]. Printed PLA and PCL scaffolds may exhibit characteristics of surface erosion or bulk degradation depending on water diffusion, polymer composition, molecular weight and scaffold architecture. Therefore, optimization of printed scaffolds requires simultaneous control of polymer chemistry and architectural parameters to achieve predictable degradation kinetics, mechanical stability and tissue regeneration outcomes.

Printed polymer implants can match anatomical defects, deliver drugs locally and support tissue regeneration. PCL, PLA and related biodegradable systems have been studied for bone, cartilage, infection control, oncology and tissue repair. Recent biomaterial developments have expanded scaffold design beyond structural support by incorporating bioactive molecules, controlled delivery strategies and multifunctional architectures aimed at improving tissue regeneration and clinical translation [75,76,77]. Bioprinting reviews highlight the device and tissue-engineering potential of printed polymers [13], porous scaffold design principles explain how architecture controls tissue ingrowth and mechanical behaviour [113], and the bone-focused 3D printing literature shows why material selection and structural design must be validated together [114]. Growth factor-releasing printed scaffolds illustrate how local bioactive delivery can be combined with regenerative architecture [115]. Recent scaffold strategies further emphasize the importance of integrating mechanical performance, degradation behaviour, biological signalling and controlled therapeutic delivery to achieve clinically meaningful regenerative outcomes [75,76]. Earlier additive manufacturing reviews of tissue and organ constructs also inform the implant/scaffold discussion [116]. The translational standard for implants is higher than for prototype scaffolds. Mechanical fatigue, sterilization, degradation kinetics, local pH, tissue integration, burst release, inflammatory response and removal or resorption must be assessed.

For combination products, the drug and device functions must be evaluated together. Although printed implants and scaffolds provide opportunities for patient-specific design and localized therapy, their clinical translation remains dependent on demonstrating predictable degradation behaviour, reproducible mechanical performance and consistent therapeutic outcomes compared with established implantable systems [76,77]. The adoption of printed implants will therefore depend on demonstrating advantages such as improved anatomical matching, enhanced therapeutic localization or reduced surgical complexity compared with conventionally manufactured implants.

### 4.5. Biologics, Cells and Living Therapeutics

Biologics and living cells favour low-temperature, water-rich and cytocompatible printing approaches because proteins, nucleic acids and cells are sensitive to thermal stress, shear forces and chemical exposure during processing. Semi-solid extrusion and hydrogel-based bioprinting can provide hydrated environments that support protein stability and cellular function; however, challenges related to storage stability, sterility, potency preservation, oxygen exposure, crosslinking effects and drying remain significant. Cell-laden constructs raise additional concerns related to cell identity, viability, potency, genetic stability and aseptic manufacturing [13,14,43,59,60]. Recent advances in bioink engineering have focused on improving print fidelity, biological compatibility and tissue-specific functionality through the development of hybrid, bioactive and stimuli-responsive polymer networks [61,63].

The performance of printed biologic and cell-based systems depends on achieving an appropriate balance between mechanical stability and biological activity. Increasing polymer concentration or crosslinking density may improve structural integrity but can restrict nutrient diffusion, cell migration and tissue maturation. Conversely, highly permissive biological matrices may exhibit insufficient mechanical strength or shape retention after printing. Therefore, bioink selection requires simultaneous consideration of rheological behaviour, cytocompatibility, degradation characteristics and functional outcomes.

For near-term clinical translation, acellular polymeric delivery systems remain more mature, while living constructs represent high-potential but high-complexity products. The major challenge is therefore not only achieving successful cell incorporation during printing but also establishing reproducible manufacturing processes, long-term biological functionality and regulatory pathways comparable to conventional advanced therapy products. Translation will require standardized characterization of bioinks, control of critical process parameters and demonstration of consistent biological performance across manufacturing batches.

## 5. Diagnostics: Printable Polymers as Fluidic, Optical, and Electrochemical Infrastructure

**Figure 2:**
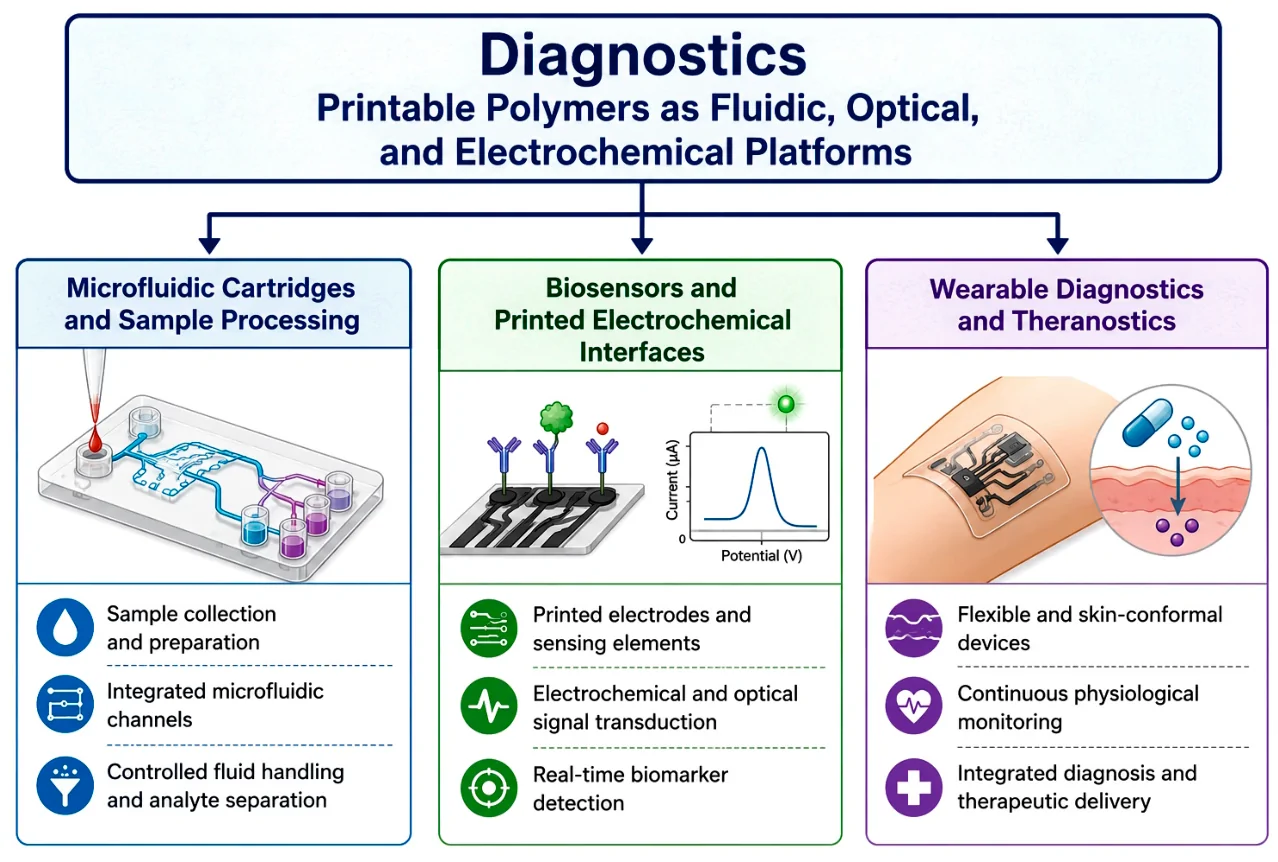
Printable polymer platforms for diagnostic and theranostic applications (created with BioRender.com).

Diagnostics based on printable polymers provide versatile fluidic, optical, and electrochemical infrastructures for advanced healthcare applications. As shown in Figure 2, created with BioRender.com (accessed on 25 May 2026), these platforms encompass microfluidic cartridges for efficient sample processing, biosensors with printed electrochemical interfaces for sensitive analyte detection, and wearable diagnostic systems integrated with theranostic functions. Such technologies provide opportunities for rapid, miniaturized and personalized disease monitoring, although clinical implementation requires further validation.

Despite the rapid progress of printable diagnostic platforms, their performance is strongly influenced by polymer composition, surface characteristics, device architecture and integration of functional components. The major advantage of polymer-based additive manufacturing lies in design flexibility, rapid customization and integration of multiple diagnostic functions [117]. However, material-induced variability, surface interactions and long-term functional stability remain important considerations when designing reliable diagnostic platforms.

### 5.1. Microfluidic Cartridges and Sample Processing

3D printing can accelerate microfluidic development by enabling rapid iteration of channels, reservoirs, mixers, filters and reaction chambers. Foundational reviews identified enablers and barriers for printed microfluidics [16] and anticipated a broader microfluidic fabrication shift [15]. More recent bioanalytical reviews show that 3D printing can support automated, reproducible, low-cost microanalytical devices when channel resolution, surface chemistry and post-processing are controlled [27]. Recent developments in microfluidic technologies have further emphasized the integration of advanced chip architectures, artificial intelligence-assisted analysis and application-specific designs for improving diagnostic performance. These advances highlight the potential of printed microfluidic platforms for laboratory medicine and biomedical applications, while also reinforcing the need for rigorous validation of fluid transport, material compatibility and analytical reliability under clinically relevant conditions [118,119]. SLA material characterization for self-driven microfluidics further illustrates the importance of cure conditions, wetting and biocompatibility for diagnostic performance [69]. Classical microfluidic transport and lab-on-a-chip perspectives remain relevant when interpreting flow, mixing and surface effects in printed diagnostic cartridges [120,121].

For diagnostic translation, device geometry should be considered together with material characterization, post-cure conditions, extraction procedures, surface treatment and assay compatibility. Surface roughness, optical clarity and leachable components can influence assay transfer and analytical performance in printed microfluidic devices [16], while recent 3D-printed microfluidic studies have emphasized the importance of bioanalytical validation and reproducibility [27]. Dedicated SLA material investigations further demonstrate that resin chemistry, post-curing conditions and capillary flow behaviour must be optimized together to achieve reliable microfluidic function [69]. Although additive manufacturing enables rapid prototyping and complex microfluidic architectures, successful implementation requires careful control of material selection, surface properties, optical compatibility and extractable components.

### 5.2. Biosensors and Printed Electrochemical Interfaces

Electrochemical biosensors benefit from printed conductive polymer composites and customizable electrode geometry. Carbon-based filaments, graphene composites, CNT-loaded inks, metal nanoparticles and conductive polymers have been used to fabricate electrodes, housings and integrated sensing platforms [83]. The electrochemical 3D-printing field was summarized early by Ambrosi and Pumera [17], and additive-manufactured analytical-device reviews later connected printed materials to sensing performance [73]. Studies on electroanalysis and additive-manufacturable biosensor electrodes show that conductivity, activation, surface area and fouling are critical variables rather than secondary processing details [46,59]. Recent advances in conductive polymer-based sensors and nanomaterial-modified electrodes have further demonstrated the importance of electrode composition, surface modification and charge-transfer characteristics in improving analytical sensitivity and selectivity [74,83,122]. Specific electrochemical examples include printed cells and sensors [123], graphene-electrode activation [124], PLA–graphene electrode activation for dopamine sensing [125] and disposable 3D-printed bioanalytical sensors [126]. Recent paper-based electrochemical biosensor platforms using conductive inks further demonstrate the potential of printable conductive materials for low-cost and decentralized diagnostic applications [84].

These examples demonstrate that polymer-based additive manufacturing can enable rapid customization of sensing interfaces; however, analytical performance remains strongly dependent on material composition, surface properties and fabrication history. Surface roughness, filler dispersion, electrical percolation, electrode activation, fouling behaviour and reagent immobilization can influence sensor response and reproducibility. Therefore, printed biosensors should be evaluated using analytical parameters such as limit of detection, linear range, selectivity, precision, stability and sample-matrix effects in addition to conventional printing characteristics. Recent studies on conductive polymer composites and printed biosensing platforms further highlight the importance of maintaining consistent electrical properties and functional stability under application-relevant conditions [85,96,127].

### 5.3. Wearable Diagnostics and Theranostics

Wearable diagnostic systems require polymers that are flexible, skin-compatible, breathable, adhesive and compatible with sweat, interstitial fluid or wound exudate. Printing can create personalized fit, microchannels, reagent reservoirs, sensor housings and mechanical interfaces. Hollow and non-transdermal microneedle reviews are relevant where the wearable interface samples interstitial fluid rather than only delivers the drug [12,29]. For electrochemical wearables, printed polymer composites must maintain electrode performance under bending, hydration and biofouling conditions, making material-focused biosensor reviews directly relevant [73,74].

Closed-loop polymeric systems must be evaluated as integrated platforms because sensor drift, false signals, actuator performance, skin interaction and material stability directly influence system reliability. Wearable polymer composites must maintain functional performance under repeated mechanical deformation, hydration exposure and biological interactions, requiring careful integration of material properties, sensor design and device architecture. The future development of polymer-based theranostic systems will depend on achieving stable sensing–actuation interfaces and robust material performance under dynamic physiological conditions.

## 6. AI-Powered 3D Bioprinting Platforms for Advanced Disease Modelling and Precision Diagnostics

### 6.1. Workflow of Disease Diagnosis Using AI-Integrated Bioprinting

**Figure 3:**
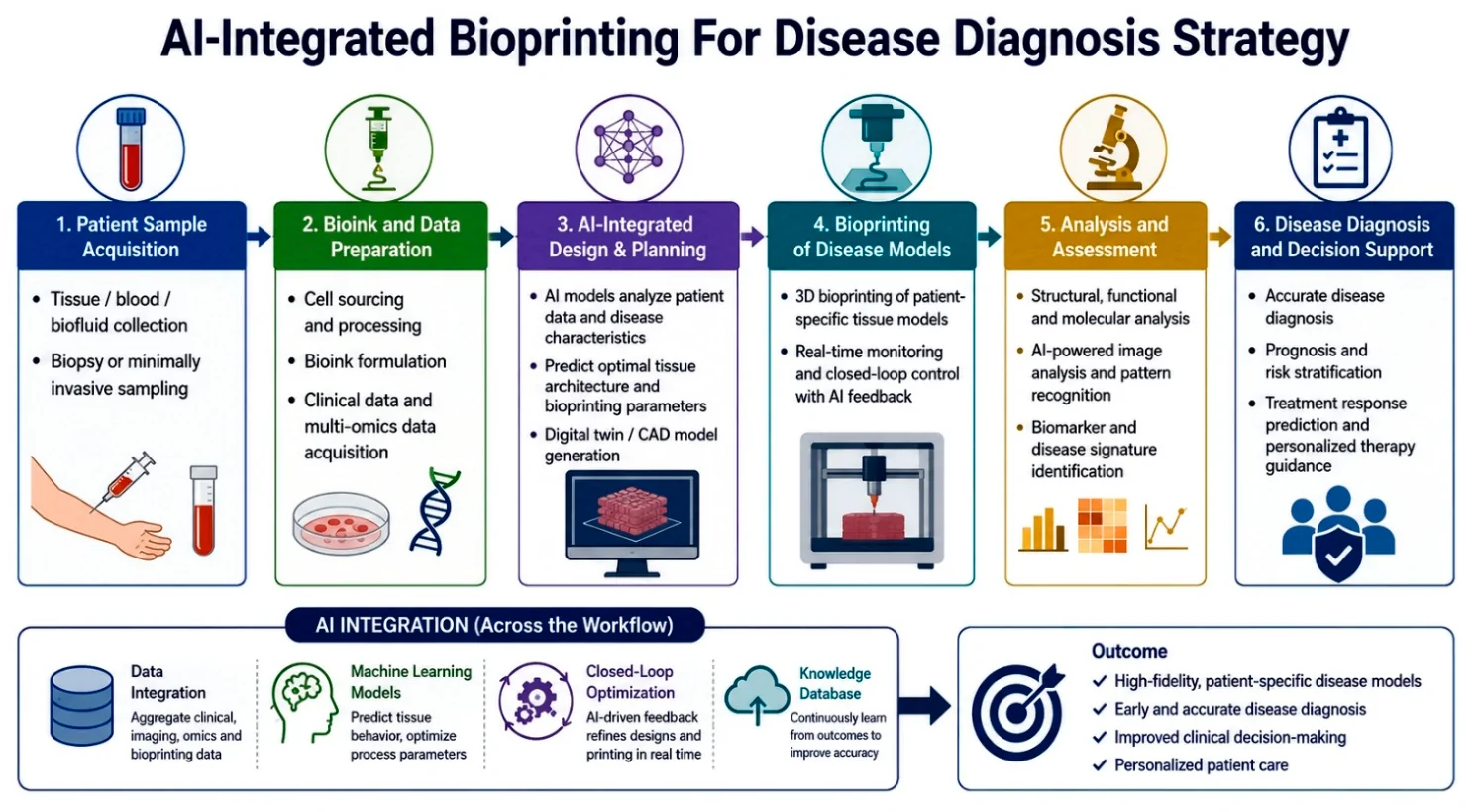
AI-integrated 3D bioprinting workflow for disease modelling and precision diagnostics (Created with BioRender.com).

The incorporation of artificial intelligence into 3D bioprinting provides an emerging framework for disease modelling and precision diagnostics. This approach integrates patient-derived biological samples, bioprinting technologies, analytical tools and computational modelling to develop disease-relevant constructs. Previous work on 3D bioprinting has shown its value for biomedical devices, tissue engineering and tissue-like model development, while AI-based approaches are increasingly being explored for the optimization of 3D-printed biomedical and pharmaceutical systems [13,14,19]. As shown in Figure 3, created with BioRender.com (accessed on 25 May 2026), the workflow begins with patient sample collection through biopsy, blood collection or other minimally invasive procedures. These samples provide cellular and molecular information that can support disease modelling and individualized clinical evaluation.

Following sample collection, patient-derived cells may be isolated, cultured and incorporated into suitable bioink formulations. Bioink selection is important because printability, cytocompatibility, shape fidelity and post-printing cell function strongly influence the quality of the printed construct [58,59]. In parallel, clinical records, imaging data, genomic information, transcriptomic profiles, proteomic data and other multi-omics datasets may be integrated to establish a broader patient profile. AI-based analysis can then support the connection between biological data and digital design requirements. Machine-learning methods may assist in identifying tissue morphology, bioink composition and printing parameters suited to specific disease models [19].

### 6.2. Digital Twins, Computer-Aided Design (CAD) Models and Predictive Optimization

Digital design tools, computer-aided design models and predictive computational approaches can be used to create virtual representations of diseased tissues before fabrication. These models may support construct design, process planning and parameter optimization. In this context, AI-assisted prediction may improve reproducibility and reduce experimental variability when supported by well-characterized datasets [19]. Patient-specific design parameters can then be translated into 3D bioprinted constructs using appropriate bioinks, printing conditions and post-processing strategies [58,59]. In materials-driven bioprinting workflows, AI models are increasingly being applied to predict polymer and bioink behaviour rather than only optimize printing geometry. Machine-learning approaches can integrate material descriptors such as polymer concentration, molecular weight, viscosity, storage modulus (G′), loss modulus (G″), crosslinking density, degradation rate and printing parameters to predict outcomes including printability, shape fidelity, mechanical stability and post-print functionality. Such models provide opportunities to establish predictive relationships between polymer characteristics, processing conditions and final construct properties [128].

For hydrogel-based systems, future AI-assisted digital models may integrate experimentally derived rheological parameters with constitutive descriptions of viscoelastic behaviour, swelling kinetics and degradation processes. However, current applications remain largely dependent on experimentally generated datasets, and comprehensive integration of polymer network mechanics, enzymatic degradation and long-term biological remodelling into predictive models remains a developing area [63,129]. Real-time monitoring systems and AI-enabled feedback mechanisms can strengthen fabrication control by assessing printing fidelity, structural accuracy, environmental conditions and bioink behaviour during the printing process. Such closed-loop approaches may reduce operator-dependent variability and improve consistency when supported by experimentally validated datasets and appropriate material descriptors [19,130,131].

Recent studies have further explored artificial intelligence-assisted prediction models for optimizing bioink composition, printing parameters and construct performance. Machine-learning approaches have been applied to predict polymer printability, identify suitable processing conditions and optimize formulation variables using datasets containing material properties, rheological behaviour, printing parameters and output characteristics [128]. Deep-learning approaches have also been investigated for image-based analysis, structural assessment and automated interpretation of complex biological constructs, while AI-integrated bioprinting frameworks aim to connect digital design, fabrication control and precision tissue engineering [129,132]. However, clinical translation of these systems requires experimentally validated datasets, independent model testing, interpretability of algorithmic decisions and confirmation that predicted improvements translate into measurable biological or therapeutic outcomes.

### 6.3. Post-Fabrication Assessment and Disease Signature Discovery

After fabrication, 3D bioprinted disease models require structural, functional and molecular characterization. Evaluation may include construct morphology, cell viability, tissue-specific function, biomarker expression and response to therapeutic exposure. AI-assisted image analysis, computational modelling and pattern-recognition methods can support the interpretation of complex datasets generated from printed biological constructs [19]. These approaches may help identify disease-associated features that are difficult to detect using conventional analytical methods alone. The identification of disease signatures may contribute to diagnostic classification, biomarker discovery and therapeutic screening. Patient-specific disease models may also support the assessment of disease progression, risk stratification and potential treatment response. The clinical value of these models depends on the biological relevance of the printed construct, the quality of the input data and the validation of the computational models used for interpretation [13,14,19].

Post-fabrication AI assessment can be extended beyond biological image interpretation by incorporating quantitative descriptors of the printed polymer construct. Parameters such as pore architecture, filament distribution, surface morphology, swelling behaviour and degradation characteristics can be integrated with imaging and molecular datasets to establish relationships between material properties and biological performance [63]. Machine-learning models have been explored to correlate polymer composition, processing parameters and construct characteristics with printing performance, enabling prediction of printability and optimization of fabrication variables [128]. AI-integrated bioprinting frameworks further highlight the potential of combining computational analysis with experimental datasets for improving construct evaluation and precision tissue-engineering workflows [129]. However, reliable prediction requires standardized characterization datasets linking polymer properties, processing history and biological outcomes, as variability in biomaterial preparation and experimental conditions remains a major limitation for model transferability.

### 6.4. Closed-Loop AI Ecosystem for Precision Medicine

A key future direction is the development of closed-loop systems in which clinical, imaging, omics and bioprinting data are continuously integrated. Machine-learning models may improve over time when trained on well-characterized datasets and validated using reproducible experimental outcomes [19]. Feedback from post-fabrication testing can also inform later decisions on construct design, bioink selection, process parameters and quality control. This approach is consistent with quality-by-design principles, where material attributes, process parameters and final product performance are linked within a defined design space [131].

The integration of bioprinting and AI may help connect tissue engineering, computational modelling and clinical decision support. Potential applications under investigation include patient-specific disease modelling, disease-response prediction, drug screening and personalized therapeutic planning. However, effective implementation of AI-assisted bioprinting will require experimentally validated datasets, interpretable computational models and standardized integration between biological information, material properties and printing parameters [13,14,19,59,131]. Therefore, AI-assisted bioprinting should be considered a complementary platform for precision medicine, with future progress depending on integration of validated material datasets, predictive models and experimentally confirmed biological outcomes.

## 7. Quality-by-Design, Characterization and Manufacturing Control

While polymer selection and printing technologies determine the technical feasibility of additive manufacturing, successful implementation depends on the ability to control material variability, manufacturing processes and final product performance. Quality-by-design approaches provide the framework linking polymer characteristics, printing parameters and clinically relevant quality attributes. The following sections discuss critical material attributes, critical process parameters and analytical strategies required for reproducible polymer-based additive manufacturing.

### 7.1. Critical Material Attributes

The translation of polymer-based additive manufacturing from laboratory-scale demonstrations to clinically relevant products requires a systematic connection between material properties, printing conditions and final product performance. Quality-by-design (QbD) provides this framework by linking critical material attributes, critical process parameters and critical quality attributes to ensure reproducible manufacturing and predictable therapeutic or diagnostic outcomes [133,134]. The critical material attributes are polymer molecular weight, polydispersity, moisture content, particle size, thermal transitions, melt viscosity, rheological behaviour, filament diameter, drug solid state, residual monomer content, photoinitiator concentration, filler dispersion, conductivity, and bioburden. The importance of these attributes is application-dependent because they directly influence critical product characteristics, including mechanical integrity, porosity, drug-release behaviour, dimensional accuracy, biological compatibility and analytical performance. Therefore, characterization should not be limited to material confirmation but should establish clear relationships between polymer properties, printing conditions and final product functionality [135,136].

Other properties, like optical transparency, autofluorescence, wetting behaviour, non-specific adsorption and compatibility with assay reagents, are also important for analytical reliability in diagnostic systems. The selection of characterization strategies should be determined by the specific risks of each printing technology and biomedical application [137,138]. Differential scanning calorimetry and thermogravimetric analysis are important thermal techniques for hot-melt extrusion, fused deposition modelling and selective laser sintering. Rheological evaluation is important for semi-solid extrusion and hydrogel-based printing, where printability and shape fidelity are strongly dependent on viscosity and shear-thinning behaviour. Spectroscopic, chromatographic and extraction studies are required for photopolymer systems to determine chemical conversion, residual components and potential leachables [95,100,139]. The microstructural and dimensional properties can be studied using scanning electron microscopy, micro-computed tomography or optical profilometry. Electrochemical impedance analysis, contact-angle measurement, leachables testing and analytical validation should supplement mechanical, dimensional and stability assessments in diagnostic platforms.

### 7.2. Critical Process Parameters and Process Analytical Technology

Critical process parameters include nozzle temperature, extrusion pressure, screw speed, feed rate, layer height, infill, shell thickness, bed temperature, laser power, scan speed, exposure dose, cure time, post-cure conditions, drying conditions and sterilization. A process is not controlled simply because it follows a digital file [44,140,141]. Variations in printing parameters can modify layer adhesion, internal porosity, surface characteristics and mechanical performance, which subsequently affect drug release, device reliability and diagnostic accuracy. Consequently, characterization strategies should integrate structural, mechanical, chemical and functional evaluation rather than relying on dimensional assessment alone. Environmental humidity, printer calibration, feedstock age and post-processing can change the final product. Process analytical technology can include filament diameter monitoring, weight checks, machine vision, in-line near-infrared (NIR) or Raman spectroscopy, thermal imaging, print-force sensing, electrical resistance measurement, image-based defect detection and dissolution modelling [142,143]. Quality-by-design reviews provide the framework for mapping critical material attributes and critical process parameters to dosage-form performance [131]. Machine-learning and AI discussions are useful when they are tied to experimentally verified process data rather than used as generic prediction claims [19].

Mechanical characterization of printed polymers remains essential for implants, microneedles, cartridges and scaffolds [130], while digital-control strategies are especially important for decentralized or point-of-care manufacture [144]. Variations in material batches, environmental conditions, printer settings and post-processing steps can significantly influence final product performance, highlighting the need for integrated process control strategies. Therefore, successful translation requires moving beyond parameter monitoring toward integrated quality systems that establish clear relationships between material attributes, process conditions and clinically relevant product outcomes. Table 4 summarizes the suggested critical quality attributes and corresponding control strategies for polymer-based 3D printing in drug delivery and diagnostic applications.

## 8. Regulatory, Clinical and Ethical Considerations

### 8.1. Regulatory Classification and Product Pathway

The quality attributes established during manufacturing control directly influence regulatory evaluation and clinical acceptance of polymer-based printed products. Regulatory classification depends on primary mode of action: a printed product may be a drug, medical device, in vitro diagnostic, biologic or combination product. FDA guidance for additively manufactured medical devices emphasizes design, manufacturing and device testing considerations and FDA discussions of point-of-care printing highlight unresolved responsibilities when production moves into hospitals or clinics [145,146]. For pharmaceuticals, the approval of porous levetiracetam establishes feasibility but does not create a universal pathway for personalized printlets [4,5]. Regulatory evaluation of printed products will depend on appropriate product-specific evidence, including demonstration of safety, performance and consistency. For diagnostic products, analytical sensitivity, specificity, precision, robustness, interference, usability and clinical performance are central. For combination products, the interaction between therapeutic and diagnostic functions must be characterized.

Recent regulatory discussions have further emphasized the need for standardized manufacturing controls, digital traceability, risk-based quality assessment and clearly defined responsibilities for decentralized and point-of-care production of additively manufactured healthcare products. These considerations are particularly important for personalized systems, where conventional batch-release strategies may require adaptation to patient-specific manufacturing workflows [143,147,148].

### 8.2. Clinical Value and Evidence Generation

Clinical adoption should be driven by unmet need. Strong candidates include pediatric dose flexibility, geriatric dysphagia, polypharmacy, rare diseases, implants matched to anatomy, microneedle vaccination or biologic delivery, and point-of-care diagnostics where rapid design iteration or local manufacturing improves access [48,98,136]. Printed products are less compelling when conventional mass manufacturing already provides safe, inexpensive, stable, and clinically adequate products. Clinical trials involving personalized point-of-care manufacturing require careful governance: who releases the batch, who verifies the design file, how deviations are handled, and how stability is justified for small-batch or patient-specific products [98,136].

Recent discussions of clinical trial frameworks for 3D-printed medicines highlight the need for predefined manufacturing controls, documentation and responsibilities [98,133,134,136]. However, clinical adoption of personalized printed products will depend on demonstrating clear advantages over conventional manufacturing approaches, including improved therapeutic outcomes, patient-specific benefits, or enhanced accessibility. Therefore, regulatory acceptance will require not only technical validation of the printing process but also evidence that personalization provides meaningful clinical value [98,133,134].

### 8.3. Ethics, Data Integrity and Cybersecurity

Digital manufacturing introduces data integrity and cybersecurity risks. A corrupted design file can become a dose error; a modified sensor algorithm can become a therapeutic hazard; and a decentralized manufacturing system can obscure accountability. Ethical issues include equitable access, data privacy, algorithmic dose selection, responsibility for adverse events and the cost of personalized manufacturing [149,150].

These questions are not separate from materials science, because the clinical value of a printable polymer platform depends on the system in which it is used [149,150]. As additive manufacturing becomes increasingly connected with digital design files, artificial intelligence, and decentralized production, ensuring data integrity, cybersecurity, and clear responsibility frameworks will become essential components of product quality and patient safety [133,134]. Therefore, ethical and regulatory considerations should be incorporated during platform development rather than addressed only after technological validation.

## 9. Sustainability and Standardization

### 9.1. Environmental Considerations

As polymer-based additive manufacturing develops toward broader biomedical use, sustainability and standardization become important considerations alongside material selection and manufacturing efficiency. Additive manufacturing can reduce tooling waste and inventory, but it can also generate failed prints, support materials, disposable cartridges, photopolymer waste and energy demand. SLS has motivated research into lower-energy or cold laser sintering approaches [23]. Sustainability should be treated as a design criterion rather than a promotional claim. Solvent-free processing, biobased polymers, recyclable cartridges, efficient post-curing and reduced failed-print rates can improve environmental performance. However, sustainability strategies in biomedical additive manufacturing must be evaluated alongside material safety, sterilization requirements, reproducibility and regulatory compliance, as reducing environmental impact cannot compromise product quality or patient safety.

Recent studies have increasingly examined sustainability in additive manufacturing by considering material selection, waste reduction, resource efficiency and process optimization. Emerging approaches include the valorization of sustainable feedstocks, recycling strategies and improved manufacturing workflows to reduce the environmental impact of printed products [87,94,141]. However, in pharmaceutical and biomedical applications, sustainability strategies must remain compatible with material safety, process reliability and intended product performance.

### 9.2. Reporting Standards and Reproducibility

Reproducibility is limited by incomplete reporting of printer model, software, slicing parameters, feedstock preparation, material lot, moisture, post-processing and environmental conditions. A minimum reporting checklist for polymer-based biomedical printing should include material identity, molecular weight or grade, drug loading, particle size or filament diameter, rheology or thermal data, print parameters, post-processing, sterilization, characterization methods and raw performance metrics. Without such detail, promising results cannot be reproduced or translated [151,152]. Therefore, standardized reporting practices are essential for meaningful comparison between studies, reliable process optimization and development of reproducible manufacturing frameworks.

## 10. Future Perspectives

The next phase of polymer-based 3D printing will be defined by integration and evidence. This progression requires simultaneous advancement in materials science, manufacturing control, regulatory frameworks and clinical validation. Mechanistic modelling and machine learning can connect polymer properties, process parameters, geometry and performance, but QbD logic should remain the foundation for defining a valid design space [153]. AI-enabled formulation and process optimization may accelerate development when models are trained on well-characterized print and release datasets [19]. Point-of-care manufacture will require digital file control, traceability and validated release procedures [144], and clinical-trial frameworks for printed medicines are beginning to define how patient-specific products can be evaluated ethically and reproducibly [154]. Multi-material printing will combine rigid supports, soft interfaces, conductive traces, responsive hydrogels and drug reservoirs. For drug delivery, high-impact opportunities include pediatric and geriatric personalization, complex polypills, microneedle vaccination, biologic delivery and long-acting local implants. For diagnostics, strong opportunities include low-cost microfluidic cartridges, wearable biochemical sensing, interstitial-fluid sampling and point-of-care analytical systems. Future advancements in AI-integrated bioprinting are expected to incorporate multimodal patient datasets, digital twin technologies, and autonomous manufacturing platforms, enabling real-time disease prediction, rapid therapeutic screening, and highly personalized precision medicine. Future theranostic platforms may integrate sensing, decision-support and therapeutic functions. Their success will depend on validated materials, robust controls and compelling clinical evidence. General additive manufacturing technology principles are also used to contextualize process selection and system design [155].

A critical comparison across current platforms indicates that no single polymer, printing technology or application strategy provides an ideal solution for all biomedical needs. Thermoplastic systems offer manufacturing maturity but are limited by thermal constraints, hydrogels provide biological compatibility but require improved mechanical stability, and photopolymer systems provide high resolution but demand rigorous chemical safety evaluation. Similarly, drug-delivery platforms have demonstrated stronger translational progress than fully integrated diagnostic and theranostic systems, which still require substantial analytical and clinical validation. Future advancement will therefore depend on selecting application-specific material–process combinations rather than pursuing universal printing solutions.

## 11. Conclusions

Additive manufacturing using polymers provides a versatile bridge between pharmaceutical technology and diagnostic engineering. Its decisive advantage is not merely shaping complexity but the ability to translate digital design into function through material chemistry, geometry and controlled process history. Programmable release, personalized dosing, multi-drug compartmentalization, microneedles, implants and local therapeutic systems in drug delivery are enabled by printed polymers. In diagnostics they enable rapid device iteration, integrated microfluidics, electrochemical and optical interfaces, wearable platforms and potential closed loop theranostics. The integration of artificial intelligence with 3D bioprinting provides an emerging approach for the creation of patient-specific disease models, increasing diagnostic accuracy, augmenting clinical decision-making, and accelerating the move towards personalized healthcare. Future progress should focus on validated polymer libraries, reproducible process windows, stability testing, extractables and leachables assessment, clinically justified personalization and regulatory alignment. With these elements in place, polymer-based 3D printing has the potential to progress from experimental prototypes toward more reliable platforms for personalized medicines, accessible diagnostics and integrated patient-specific healthcare.

## Figures and Tables

**Table 1:** Physicochemical and rheological characteristics of polymers relevant to FDM and HME-based biomedical printing.

Polymer System	Tg/Thermal Transition	Rheological and Processing Considerations	Biomedical Printing Relevance	References
PLA and other thermoplastic polymers	Tg ~55–65 °C; melting behaviour, crystallinity and thermal history influence mechanical properties and processing suitability	Melt viscosity, thermal history, cooling behaviour and process parameters influence filament formation, interlayer adhesion and dimensional accuracy during FDM	Pharmaceutical filaments, oral dosage forms, implants and structural biomedical components	[42,44,45,46]
PCL (polycaprolactone)	Low Tg (~−60 °C) and low melting temperature (~58–62 °C); semicrystalline biodegradable polyester	Low-temperature extrusion enables processing of thermosensitive systems; molecular characteristics and blend composition influence flow behaviour and mechanical performance	Biodegradable implants, scaffolds and long-term drug-delivery systems	[39,47]
PVA (polyvinyl alcohol)	Tg ~65–85 °C; moisture content strongly affects thermal and mechanical behaviour	Moisture-dependent viscosity, filament flexibility and extrusion behaviour influence printability and reproducibility	Oral printlets, soluble matrices and personalized dosage forms	[6,48,49,50]
PVP/PVPVA-based polymers	Amorphous pharmaceutical polymers with grade-dependent thermal behaviour and strong drug–polymer interactions	Polymer morphology, rheological behaviour, plasticization and miscibility influence hot-melt extrusion stability and solid-state characteristics	Amorphous solid dispersions and extrusion-based drug-delivery systems	[51,52]
Eudragit^®^ copolymers	Amorphous methacrylate-based pharmaceutical polymers with grade-dependent thermal transitions, solubility and swelling characteristics	Polymer composition and extrusion conditions influence flow behaviour, polymer–drug interaction and release performance	Modified-release and enteric pharmaceutical printing applications	[53,54]
Polymer blends and multicomponent systems	Polymer blending modifies thermal transitions, miscibility, crystallinity and mechanical behaviour	Phase interactions, plasticization effects and rheological tuning determine extrusion processability and product performance	Tailored biomedical printing platforms requiring optimized processing windows	[46,55]

**Table 2:** Polymer classes and translational considerations in polymer-based 3D printing for drug delivery and diagnostics.

Polymer/Material Class	Representative Examples	Drug Delivery Relevance	Diagnostic Relevance	Key Translational Risks	References
Thermoplastics	PVA, PLA, PCL, EVA, cellulose derivatives, polymethacrylates	Oral printlets, implants, reservoirs, scaffolds; geometry- and diffusion-controlled release	Housings, holders, low-cost cartridges, fixtures	Thermal stress, filament brittleness, moisture sensitivity, crystallization, batch variability	[42,43,45,48,52]
Biodegradable polyesters	PLA, PCL, PLGA-related systems	Localized depots, tissue scaffolds, long-acting implants	Bioabsorbable sampling interfaces and implantable sensors	Autocatalytic degradation, acidic microenvironments, nonlinear release-degradation coupling	[39,40,75,76,77]
Hydrogels/natural polymers	Alginate, gelatin, chitosan, hyaluronic acid, cellulose derivatives	Biologics, cells, wound dressings, microneedles, responsive release	Bioinks, reagent matrices, sample interfaces, tissue-facing sensors	Microbial burden, swelling, drying, weak mechanics, batch variability	[61,62,63,64,78]
Photopolymers	PEGDA, methacrylates, urethane acrylates, thiol-ene resins	High-resolution microneedles, micro-implants, lattice depots	Microfluidics, optics, chips, sensor substrates	Residual monomer/photoinitiator, incomplete conversion, extractables/leachables	[68,69,79,80,81,82]
Elastomers	Silicone, TPU, soft acrylate networks	Flexible patches, reservoirs, seals, tissue-matched implants	Wearables, valves, pumps, skin-conformal devices	Drug/reagent adsorption, sterilization effects, interfacial adhesion	[72]
Conductive/nanocomposite polymers	Carbon, graphene, CNTs, MXenes, metals, magnetic particles	Triggered release, antimicrobial or imaging functions	Electrochemical sensors, capture systems, electrodes, optical/electrical transduction	Dispersion, percolation variability, cytotoxicity, fouling, regulatory complexity	[74,83,84,85]

**Table 3:** Printing technologies, biomedical fit and quality risks.

Technology	Typical Polymer/Feedstock	Strengths	Constraints	Best-Fit Applications	References
FDM/HME	Drug-loaded thermoplastic filaments	Low cost, digital geometry, robust solid parts	Heat and shear stress, filament feeding, limited resolution	Oral printlets, implants, housings	[42,44,45,48,52]
Semi-solid extrusion	Hydrogels, pastes, gels, lipid/polymer inks	Low temperature, high loading, multi-material deposition	Rheology, drying, shrinkage, lower resolution	Polypills, biologics, bioinks, wound systems	[57,58,61,63,95]
Direct powder extrusion	Powder blends processed directly	Avoids filament manufacture; flexible feedstocks	Powder feeding, mixing, residence-time control	On-demand personalized solid dosage forms	[24,91,92]
SLA/DLP/CLIP	Photopolymer resins	High resolution, smooth surfaces, arrays	Residual photochemistry, cure depth, resin biocompatibility	Microneedles, microfluidics, sensor substrates	[68,79,80,81,82]
SLS	Polymer/drug powders	No solvent/binder, porous structures, no filament	Thermal exposure, powder flow, laser optimization	Oral printlets, porous depots, scaffolds	[25,74,93]
Binder jetting	Powder bed plus binder	High porosity, rapid disintegration, high dose potential	Powder-bed uniformity, binder migration	Fast-dispersing tablets	[5,52]
Inkjet/aerosol jet	Drug solutions, conductive inks, bioactive inks	Precise patterning and electronics integration	Viscosity and clogging limits, drying artefacts	Biosensors, reagent deposition, thin films	[84,96]

**Table 4:** Suggested critical quality attributes and control strategies for polymer-based 3D printing in drug delivery and diagnostic applications.

Product Type	Critical Quality Attributes	Common Failure Modes	Recommended Controls	References
Oral printlets	Dose, content uniformity, dissolution, disintegration, friability, stability, patient acceptability	Thermal degradation, filament variability, incomplete fusion, dose error	Design-space mapping, in-line mass/NIR checks, dissolution modelling, stability studies	[45,48,52,103,104,131]
Microneedles	Needle height, tip radius, insertion force, fracture force, drug load, dissolution/swelling, sterility	Needle fracture, incomplete insertion, residual photochemistry, backing failure	Mechanical insertion tests, skin models, extraction, biocompatibility, sterilization validation	[11,29,71,136]
Implants/scaffolds	Mechanical strength, fatigue, degradation, release, sterility, tissue response	Burst release, acidic degradation, inflammatory reaction, mechanical failure	Micro-CT, fatigue testing, degradation-release coupling, in vivo relevance	[39,75,76,77]
Microfluidic diagnostics	Channel geometry, wetting, optical clarity, flow rate, limit of detection, specificity, reagent stability	Blocked channels, leachables, nonspecific adsorption, evaporation	Dimensional metrology, flow testing, surface treatment, extraction and analytical validation	[27,118,119]
Wearable theranostics	Sensor accuracy, adhesion, actuation safety, comfort, data integrity, cybersecurity	Sensor drift, false trigger, skin irritation, software failure	Closed-loop validation, usability studies, biocompatibility and software lifecycle controls	[72,83,84,96,138]

## Data Availability

No new data were created or analyzed in this study. Data sharing is not applicable to this article.

## Abbreviations

The following abbreviations are used in this manuscript:

CLIP	Continuous liquid interface production
CNTs	Carbon nanotubes
DLP	Digital light processing
DPE	Direct powder extrusion
EVA	Ethylene-vinyl acetate
FDA	Food and Drug Administration
FDM	Fused deposition modelling
HME	Hot-melt extrusion
Micro-CT	Micro-computed tomography
MXenes	Two-dimensional transition-metal carbides, nitrides or carbonitrides
NIR	Near-infrared spectroscopy
PCL	Polycaprolactone
PEG	Polyethylene glycol
PEGDA	Polyethylene glycol diacrylate
PLA	Poly (lactic acid)
PLGA	Poly (lactic-co-glycolic acid)
PVA	Poly (vinyl alcohol)
QbD	Quality by design
SLA	Stereolithography
SLS	Selective laser sintering
TPU	Thermoplastic polyurethane
